# Reliability analysis using an exponential power model with bathtub-shaped failure rate function: a Bayes study

**DOI:** 10.1186/s40064-016-2722-3

**Published:** 2016-07-13

**Authors:** Romana Shehla, Athar Ali Khan

**Affiliations:** Department of Statistics and Operations Research, Aligarh Muslim University, 202002 Aligarh, India

**Keywords:** Bayesian, Exponential power distribution, R, Laplace approximation, LaplacesDemon, Marginal posterior density

## Abstract

Models with bathtub-shaped hazard function have been widely accepted in the field of reliability and medicine and are particularly useful in reliability related decision making and cost analysis. In this paper, the exponential power model capable of assuming increasing as well as bathtub-shape, is studied. This article makes a Bayesian study of the same model and simultaneously shows how posterior simulations based on Markov chain Monte Carlo algorithms can be straightforward and routine in R. The study is carried out for complete as well as censored data, under the assumption of weakly-informative priors for the parameters. In addition to this, inference interest focuses on the posterior distribution of non-linear functions of the parameters. Also, the model has been extended to include continuous explanatory variables and R-codes are well illustrated. Two real data sets are considered for illustrative purposes.

## Background

In reliability analysis, hazard rate plays an indispensable role to characterize life phenomena. Technically, failure or hazard rate represents the propensity of a device of age *t* to fail in the small interval of time *t* to $t + \mathrm {d}t$. The parametric models, such as gamma, Weibull, and truncated normal distributions, which are commonly used lifetime distributions display monotone failure rates. However, many physical phenomena exhibit failure rates which are non-monotonic. For example, the failure pattern of many mechanical and electronic components comprise of three stages: initial stage (or burn-in) where failure is high at the beginning of the product life cycle due to design and manufacturing problems, and decreases towards a constant level, the middle stage with an approximately constant failure rate, which is followed by a final stage (or wear-out phase), from where the failure rate starts to increase. Such failure rates are usually termed as bathtub (BT) or U shaped. The aforementioned models which allow only monotone failure rates are unable to produce bathtub curves and thus cannot adequately interpret data with this character. Bathtub models are possibly more realistic models than monotone failure rate models. Several models have been proposed one by one to model the real data with bathtub-shaped failure rate since 1980s (see Aarset 1987; Xie et al. 2002; Gupta et al. 2008 for detailed discussion). There are number of papers discussing several flexible distributions with more than two parameters, which can accommodate increasing, decreasing, unimodal and bathtub-shaped hazard functions (see, for examples Mudholkar et al. 1996; Pham and Lai 2007; Carrasco et al. 2008). Nevertheless, from the practical point of view, it is always important to consider parsimonious models with as few parameters as possible.

An interesting two-parameter lifetime model capable of producing increasing as well as bathtub hazard curve is exponential power-distribution introduced by Smith and Bain (1975). This model has been discussed by several authors not only in the context of reliability literature (for examples, Rajarshi and Rajarshi 1988 and Leemis 1986) but also within asymmetric distributions; see, for example, Delicado and Goria (2008). This model may be useful in certain cases where the product may be quite reliable and possibly even improve for some period of time, and then fail rather quickly after it begins to wear-out. A notable amount of work has been done on this model from the frequentists perspective. For example, Smith and Bain (1975) considered least squares-type estimators for the model parameters and performed Monte Carlo simulation to obtain their distributions in order to get inference results for the reliability. Koh and Leemis (1989) developed statistical procedures for maximum likelihood and least squares estimation of the parameters for the complete as well as Type-II censored data. Chen (1999) proposed an exact statistical test for the shape parameter of the model and found an exact confidence interval for the same parameter. Srivastava and Kumar (2011) presented the exponential power distribution as a software reliability model and carried out the Bayesian analysis in OpenBUGS using informative priors (gamma priors) for the parameters but didn’t consider censoring mechanism. To the best of our knowledge, regression modeling of the exponential power distribution has not yet been discussed. This article analyzes the model in the Bayesian framework assuming weakly-informative priors for the model parameters for both the complete and censored reliability data. A distinguishing feature of this paper is that the whole analysis is done in R (R Core Team 2015) and codes developed are well illustrated. Both the analytic and simulation-based Bayesian studies are conducted.

## Exponential power distribution

The exponential power (EP) model with shape parameter $\gamma >0$ and scale parameter $\alpha >0$ is defined by the following probability density function (pdf)

1 $$\begin{aligned} f(t)=\frac{\gamma }{\alpha ^\gamma }t^{\gamma -1}\,\text {exp}\bigg (\frac{t}{\alpha }\bigg )^\gamma \,\text {exp}\bigg [1-\text {exp}\bigg (\frac{t}{\alpha }\bigg )^\gamma \bigg ] \end{aligned}$$

**Fig. 1:**
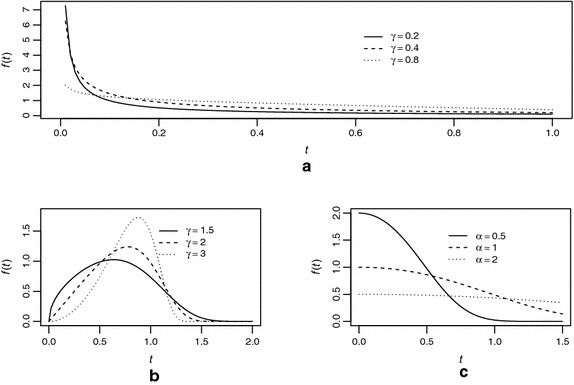
Probability density function *f*(*t*) assuming different parameter values. **a** α = 1, 0 < γ < 1, **b** α = 1, γ > 1, **c** γ = 1

This function can exhibit different behaviours depending on the values of the parameters chosen, as shown in Fig. 1.

The corresponding reliability and failure rate function of this distribution are:

$$\begin{aligned} R(t)& = \text {exp}\bigg [1-\text {exp}\bigg (\frac{t}{\alpha }\bigg )^\gamma \bigg ]\\ h(t) & = \frac{\gamma }{\alpha ^\gamma }t^{\gamma -1}\,\text {exp}\bigg (\frac{t}{\alpha }\bigg )^\gamma \end{aligned}$$

This distribution may be thought of as a truncated extreme-value distribution with a Weibull type parameterization rather than the usual location-scale parameterization (Smith and Bain 1975).

### Characterization of failure rate function

The role of the parameter $\gamma$ in determining different shapes of the failure rate function can be studied under two situations:

#### *Case 1:*

$$\gamma \ge 1$$

i For any $t>0, h'(t)>0$, thus, *h*(*t*) is an increasing function.
ii $$h(t)\rightarrow +\infty $$ as $$t \rightarrow +\infty $$.

#### *Case 2:*

$$0<\gamma <1$$

i Letting $h'(t_0)=0$, we obtain $t_0=\alpha \bigg (\frac{1-\gamma }{\gamma }\bigg )^{\frac{1}{\gamma }}$. It is evident that when $0<\gamma <1, t_0$ exists and is finite. For $t<t_0, h(t)$ is decreasing while it is increasing for $t>t_0$ showing a bathtub shaped property.
ii $h(t) \rightarrow \infty$ for $t \rightarrow 0$ and $t \rightarrow +\infty$.

**Fig. 2:**
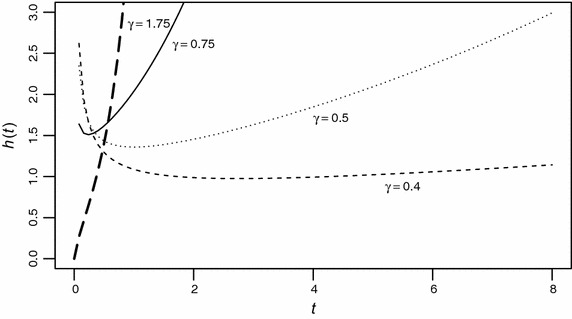
Plot of the failure rate function *h*(*t*) with $\alpha =1$ and $\gamma$ changing from 0.4 to 1.75. It is evident that the hazard function for $\gamma <1$ assumes a bathtub shape while for $\gamma \ge 1$, it increases exponentially

The bathtub character of the hazard function of EP model is depicted in Fig. 2. This model can be useful alternative to Weibull distribution for modeling lifetimes because of the three properties which it possesses. Firstly, the EP hazard function increases exponentially for large *t*, while the Weibull hazard function increases polynomially. Secondly, the hazard rate of EP model assumes a bathtub shape whereas, Weibull hazard does not. Third, the cumulative distribution of EP model is invertible, so $t=\alpha \,[\log (1-\log (1-u))]^{1/\gamma }$, where *u* is uniformly distributed between 0 and 1 and can be used to generate random variates for Monte Carlo simulation studies.

## Model formulation

The Bayesian analysis of concerned reliability model begins with the specification of likelihood function. For this, let us assume that $\underline{t}: t_1, t_2, \ldots , t_n$ be the observed lifetimes from exponential power model with pdf (1). The corresponding likelihood function can be defined as

2 $$\begin{aligned} p(\underline{t}\,|\,\gamma ,\alpha )= \bigg (\frac{\gamma }{\alpha ^\gamma }\bigg )^n\prod _{i=1}^n\big (t_i\big )^{\gamma -1}\text {exp} \bigg [\sum _{i=1}^n\bigg ({\frac{t_i}{\alpha }}\bigg )^\gamma \bigg ]\text {exp}\bigg (n-{\sum _{i=1}^n}e^{(t_i/\alpha )^\gamma }\bigg ) \end{aligned}$$

The next step in Bayesian statistics is to choose a prior distribution that expresses uncertainty about the parameters of the model before the data is observed. We considered an independent and non-informative (weakly informative) prior distributions for the parameters. Both the positive parameters are assumed to be half-Cauchy distributed according to their hyperparameters, $\text {scale}=25$ and are denoted by

3 $$\begin{aligned} \begin{aligned} \gamma \sim \text {half-Cauchy}(25)\\ \alpha \sim \text {half-Cauchy}(25) \end{aligned} \end{aligned}$$

The half-Cauchy distribution with $\text {scale}=25$ is a recommended, default, weakly informative prior distribution for a scale parameter (Polson and Scott 2012). Here, we will be using it for the shape parameter as well.

Thus, by Bayes’ rule, the joint posterior distribution can be obtained as

4 $$\begin{aligned}&p(\gamma , \alpha \,|\,\underline{t}) \propto \bigg (\frac{\gamma }{\alpha ^\gamma }\bigg )^n \prod\limits_{{i=1}}^{{n}} {\big (t_i\big )^{\gamma -1}} \text {exp}\left[\sum\limits_{{i=1}}^{{n}} {\bigg (\frac{t_i}{\alpha }\bigg )^\gamma} - \sum\limits_{{i=1}}^{{n}} {e^{(t_i/\alpha )^\gamma }}\right.\nonumber \\&\qquad \left. -\text {log}((\gamma ^2+(25)^2)(\alpha ^2+(25)^2))\right] \end{aligned}$$

In Bayesian inference, the target distribution is usually a marginal posterior distribution. Assuming $C^{*}$ to be the normalizing constant of the joint posterior distribution, the marginal posterior distributions of $\gamma$ and $\alpha$ can be obtained as

5 $$ p(\gamma \,|\,\underline{t}) =  \frac{C^{*}{\gamma ^n}\,{\prod _{i=1}^n\big (t_i\big )^{\gamma -1}}}{\gamma ^2+(25)^2} \int_0^{\infty }\alpha^{-n\gamma }\,{\text {exp}\left[\sum\limits_{{i = 1}}^{n} {\left( {\frac{{t_{i} }}{\alpha }} \right)^{\gamma } } -\text {log}(\alpha ^2+(25)^2)\right]} \times {\text{exp}}\left[-{ \sum\limits_{{i = 1}}^{n} {e^{{(t_{i} /\alpha )^{\gamma } }} }}\right]\mathrm {d}\alpha$$

and

6 $$ p(\alpha \,|\,\underline{t}) =   \frac{C^{*}}{\alpha ^2+(25)^2} \int _0^{\infty }\left(\frac{\gamma }{\alpha ^\gamma }\right)^n\prod _{i=1}^n(t_i)^{\gamma -1}\,{\text{exp}\left[\sum _{i=1}^n\left({\frac{t_i}{\alpha }}\right)^\gamma -\text {log}(\gamma ^2+(25)^2)\right]}\times {\text {exp}}\left[-{ \sum_{{i = 1}}^{n} {e^{{(t_{i} /\alpha )^{\gamma } }} }}\right]{\hbox{d}}{\gamma}$$

It can be seen that the above expressions cannot be expressed in nice closed form. Numerical intractability further becomes intense when one attempts to obtain the posterior inference for any arbitrary function of the parameters and in that case, asymptotic approximation methods (such as Lindley approximation and Laplace approximation) and simulation techniques are the only alternatives to the difficulties associated with the marginalization of the posterior densities. Lindley approximation proposed by Lindley (1980) results with enough accuracy but as Lindley points out, the required evaluations of the third derivatives of the posterior can be rather tedious, particularly, in problems with several parameters (Tierney and Kadane 1986). Contrary to Lindley (1980), the technique of Laplace approximation needs only up to second-order derivatives of the posterior and seems to be more accurate than all other conventional approximations for a range of problems. Sometimes, when the posterior is far too complex, the simulation technique has a clear advantage over asymptotic approximation methods as it does not require deep knowledge of calculus or numerical analysis. However, Laplace approximation can provide good starting points for the implementation of iterative simulation algorithms (Gelman et al. 2004) thereby, resulting in their faster convergence. Nowadays, a growth of interest can be seen in Markov chain Monte Carlo (MCMC) methods for general Bayesian calculations (see for example, Upadhyay et al. 2001; TerBraak 2006). Although, these methods are generally used in solving high-dimensional problems, we shall show that such methods can be employed straightforwardly for Bayes reliability calculations under all relatively simpler models like gamma, Weibull, exponential power model etc. There are many variants of the original Metropolis-Hastings algorithm, of which the one which has been employed in this article is the *Independence sampler*.

## Independence Metropolis algorithm

Proposed by Hastings (1970) and popularized by Tierney (1994), the independence sampler is a Metropolis- Hastings algorithm where the proposal distribution does not depend on the previous state or iteration of the chain.

$$\begin{aligned} q(\theta ^{*}|\theta ^{(s-1)})=q(\theta ^*) \end{aligned}$$

where, $\theta$ is the parameter vector. The algorithm still results in a Markov Chain despite this independence property through the definition of the acceptance probability of each new value.

Suppose we wish to simulate a sample of size *S* from a posterior density $p(\theta |y)$. The independence Metropolis (IM) algorithm can be described by the following iterative steps; where $\theta ^{(s)}$ is the vector of generated values in *s*th iteration of the algorithm:

1. Select a starting value of the chain $\theta ^{(0)}$.
2. For $s=1, \ldots , S$, repeat the following steps
  set $$\theta =\theta ^{(s-1)}$$
  generate a new parameter value, i.e. a proposal $\theta ^*$, from a proposal distribution $q(\theta ^*)$.
  calculate acceptance probability as the ratio
    $$\begin{aligned} \alpha =\text {min}\big (1, \frac{{p(\theta ^*|y)}{q(\theta )}}{{p(\theta |y)}{q(\theta ^*)}}\big ) \end{aligned}$$
  update $\theta ^{(s)} = \theta ^{*}$ with probability $\alpha$; otherwise set $\theta ^{(s)} = \theta$.

According to Ntzoufras (2009), the independence sampler is efficient when the proposal $q(\theta )$ is a good approximation of the target distribution $p(\theta |y)$. Good independent proposal densities can be based on Laplace approximation. Thus, a generally successful proposal can be obtained by a multivariate normal distribution and is given by

$$\begin{aligned} q(\theta )=\text {N}_d\big (\hat{\theta },\,\big [H(\hat{\theta })\big ]^{-1}\big ) \end{aligned}$$

where $\hat{\theta }$ is the posterior mode and can be evaluated by any efficient optimization algorithm. The quantity $H(\hat{\theta })$ is the negative of hessian matrix evaluated at posterior mode $\hat{\theta }$.

## Laplace approximation

The influence of prior distribution on posterior inferences decreases as the sample size *n* increases. These ideas are sometimes referred to as asymptotic theory. The large sample results are not actually necessary for performing Bayesian data analysis but are often useful for quick references and as starting points for iterative simulation algorithms (Gelman et al. 2004). A remarkable method of asymptotic approximation is the Laplace approximation (Tierney and Kadane 1986; Tierney et al. 1989) which accurately approximates the unimodal posterior moments and marginal posterior densities in many cases. A brief and informal description of Laplace approximation method is as follows:

Suppose $-h(\theta )$ is a smooth, bounded and unimodal function with a maximum at $\hat{\theta }$ where $\theta$ is a scalar and we wish to evaluate the integral

7 $$\begin{aligned} I=\int q(\theta )\,\text {exp}(-nh(\theta ))\,\mathrm {d}\theta , \quad \theta \in \Theta \end{aligned}$$

As presented in Mosteller and Wallace (1964), the Laplace’s method involves the Taylor’s series expansion of *q* and *h* about $\hat{\theta }$. As $h'(\hat{\theta })=0$, it follows that

8 $$\begin{aligned} h(\theta )&=h(\hat{\theta })\,+\,(\theta -\hat{\theta })'h'(\hat{\theta })\,+\,\frac{1}{2}(\theta -\hat{\theta })^2\,h''(\hat{\theta })\,+\,\ldots \nonumber \\&=h(\hat{\theta })\,+\,\frac{1}{2}(\theta -\hat{\theta })^2\,h''(\hat{\theta })\,+\,\ldots , \nonumber \\ q(\theta )&=q(\hat{\theta })\,+\,\frac{1}{2}(\theta -\hat{\theta })^2q''(\hat{\theta })\,+\,\ldots \end{aligned}$$

Substituting equation (8) in  (7), the integral *I* is approximated by

9 $$\begin{aligned} I \approx (2\pi )^{1/2}n^{-1/2}\sigma \,q(\hat{\theta })\,\text {exp}[-nh(\hat{\theta })] \end{aligned}$$

where $$\sigma =\bigg [\frac{\partial ^2h}{\partial \theta ^2}\bigg |_{\hat{\theta }}\bigg ]^{-1/2}$$.

To calculate moments of posterior distributions, we need to evaluate expressions such as:

10 $$\begin{aligned} E\{g(\theta )\}=\frac{\int g(\theta )\,\text {exp}\{-nh(\theta )\}\mathrm {d}\theta }{\int \text {exp}\{-nh(\theta )\}\mathrm {d}\theta } \end{aligned}$$

where $$\exp \{-nh(\theta )\}=L(\theta |y)\,p(\theta )$$ (Tanner 1996)

Upon applying  (9) to both the numerator and denominator of  (10) separately (with *q* equal to *g* and $q=1$), a first-order approximation

$$\begin{aligned} E\{g(\theta )\}=g(\hat{\theta })\big \{1+O(n^{-1})\big \} \end{aligned}$$

easily emerges. Thus, Laplace approximation is of order $O(n^{-1})$ uniformly on any neighbourhood of the mode. This means that it should provide a good approximation in the tails of the distribution also (e.g., Tierney and Kadane 1986; Tierney et al. 1989).

## Bayesian computation with R

There are significant number of packages contributing to the Comprehensive R Archive Network (CRAN) such as MCMCpack (Martin et al. 2013), arm (Gelman et al. 2015), LearnBayes (Albert 2014) that provide tools for Bayesian inference. But, these packages are not flexible enough to handle high-dimensional problems and at the same time, the censoring mechanism which is the most important feature of reliability data. This paper presents the contributed R package LaplacesDemon that facilitates multi-dimensional Bayesian inference and is freely available at http://www.bayesian-inference.com/software. The MCMC algorithms in LaplacesDemon are generalizable and robust to correlation between variables or parameters.

The package LaplacesDemon (Statisticat LLC 2015) not only facilitates the implementation of simple as well as more advanced simulation algorithms though the function LaplacesDemon, but also provides a function LaplaceApproximation for Laplace approximation, that evaluates objective function many times, per iteration thus, making MCMC algorithms faster per iteration (see, for example, Shehla and Khan 2013). Although, this package is not specifically meant for reliability data analysis, we have developed codes in it to deal with uncensored and censored reliability data problems.

### The function LaplaceApproximation

The function LaplaceApproximation deterministically maximizes the logarithm of the unnormalized joint posterior density using one of the several optimization techniques. The aim of LaplaceApproximation is to estimate posterior mode and variance of each parameter. Currently, this function offers 19 optimization algorithms. The function LaplaceApproximation is also typically faster because it is seeking point-estimates, rather than attempting to represent the target distribution with enough simulation draws. Another striking feature of this function is that it carries the possibility of drawing independent samples through sampling importance resampling technique via one of its arguments sir. A short length discussion of its arguments are as follows:

LaplaceApproximation(Model, parm, Data, Interval=1.0E-6,

Iterations=100, Method="SPG", Samples=1000, CovEst="Hessian",

sir=TRUE, Stop.Tolerance=1.0E-5, CPUs=1, Type="PSOCK")

where Model receives the model from a user-defined function. The argument parm requires a vector of initial values for the parameters for optimization. The argument Data accepts a listed data object on which the model is to be fitted. The argument sir takes a logical value to specify whether sampling importance resampling is to be implemented or not. It is implemented via SIR function of this package which draws independent posterior samples.

### The function LaplacesDemon

Given data, a model specification, and initial values, LaplacesDemon maximizes the logarithm of the unnormalized joint posterior density with Markov chain Monte Carlo (MCMC) algorithms, also called samplers, and provides samples of the marginal posterior distributions, deviance and other monitored variables. The function LaplacesDemon offers 41 MCMC algorithms for numerical approximation in Bayesian inference. The default algorithm is “Metropolis-within-Gibbs (MWG)”. The arguments of this function are as follows:

LaplacesDemon(Model, Data, Initial.Values, Covar=NULL,

Iterations=10000, Status=100, Thinning=10, Algorithm="MWG",

Specs=NULL, LogFile="", ...)

where Model receives the same user-defined model, Data stands for the listed data object. The argument Initial.Values requires a vector of initial values equal in length to the number of parameters. However, if Laplace approximation has been performed, the results obtained are input as initial values in this function. The argument Covar receives a $d\times d$ proposal covariance matrix (where *d* is the number of parameters) as returned by the function LaplaceApproximation. If NULL, it indicates that variance vector or covariance matrix has not been specified, so the algorithm will begin with its own estimates. The argument Iterations specifies the number of iterations that LaplacesDemon will update the parameters searching for target distribution and Status is reported after every 100 iterations. Thinning is performed via the argument Thinning to reduce autocorrelation and the number of marginal posterior samples. The argument Specs=NULL is default argument, and accepts a list of specifications for the MCMC algorithm declared in the Algorithm argument.

## Bayesian analysis of exponential power model

For fitting the exponential power model, we simulate a data set under the same model, so that the codes developed can directly be assessed. A data set of length 15 for fixed values of $\gamma =1$ and $\alpha =30$ is simulated from the exponential power model, using the self-developed function rexp.power in R and displayed in Table 1.

**Table 1:** Simulated dataset from exponential power model

8.068	11.464	18.465	36.609	6.094	35.695
40.787	21.987	20.672	1.854	6.226	5.325
23.125	11.855	27.114			

We now proceed for the posterior analysis of the model in R, which essentially requires the creation of data, model building and choosing initial values for the parameters. Before applying the independence-Metropolis algorithm to approximate the posterior density, an attempt is made to approximate it using Laplace approximation. For that, we progress by the following steps:

### Creation of data

The simulated data set is rounded up to two decimal places and entered into R using the concatenation function c and is assigned the name y. The function LaplaceApproximation requires data in a listed format. 
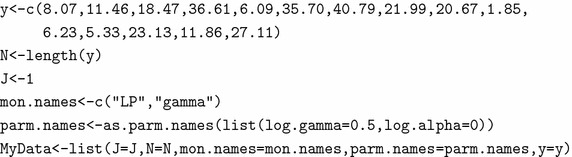
J=1 depicts that there is no regressor in the model. The object mon.names contains the variable names to be monitored. Each parameter must have a name specified in the vector parm.names, and parameter names must be included with the data using a function called as.parm.names. Finally, all these objects are combined in a list and assigned the name MyData.

### Model specification

For the exponential power error model, the likelihood is specified as in Equation (2). It is a fact that working on the log scale makes the computation numerically more stable. Thus, we define the log-likelihood as

11 $$\begin{aligned} \log {p(\underline{t}\,|\,\gamma ,\alpha )} & = n\log {\gamma }-n\gamma \log {\alpha }+(\gamma -1) \sum\limits_{{i = 1}}^{n} {\log (t_{i} )}  \nonumber \\& \quad + \sum\limits_{{i = 1}}^{n} {\left( {\frac{{t_{i} }}{\alpha }} \right)^{\gamma } }   +n- \sum\limits_{{i = 1}}^{n} {e^{{(t_{i} /\alpha )^{\gamma } }} }   \end{aligned}$$

Taking the log of the prior densities, the logarithm of the unnormalized joint posterior density is calculated according to the Bayes’ rule as:

$$\begin{aligned} \log p(\gamma ,\alpha \,|\,\underline{t}) \propto \log {p(\underline{t}|\gamma ,\alpha )}+\log {p(\gamma )}+\log {p(\alpha )} \end{aligned}$$

To get the correct posterior inference for the *positive* parameters in the situation that involves optimization of the log-posterior, is itself a difficult numerical problem. The package LaplacesDemon favours unconstrained parameterization by making log transformation of the positive parameters.

To specify the model in R, we created a function called Model as: 
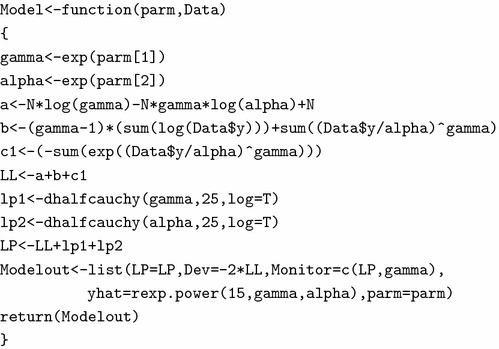
 The parameters $\gamma$ and $\alpha$ are necessarily positive, so log transformation is used for both of them to be real-valued. Thus, they are allowed to range from $-\infty$ to $+\infty$ and are transformed back in the Model function with the antilog function exp, which restores their positiveness. Having done that, LaplacesDemon may decrease log(beta) and log(alpha) viz. parm[1] and parm[2] respectively below zero without violating their half-Cauchy distribution assumptions. The logarithm of the unnormalized joint posterior density is calculated as LP, the deviance Dev, a vector Monitor of any variable desired to be monitored besides the parameters, yhat or replicates of y and the parameter vector parm and finally all of these are returned by the function Model in the form of listed data object called Modelout.

### Initial values

LaplacesDemon requires a vector of initial values, each element of which is a starting point for the estimation of model parameters. Setting all of the parameters equal to zero, is a safe choice. The user may also use GIV (generate initial values) function which randomizes each initial value, in the absence of any prior knowledge about the parameter 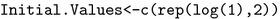


### Approximation by Laplace’s method

**Fig. 3:**
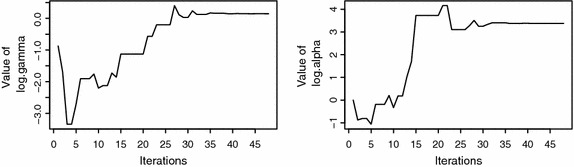
Plots depicting the convergence of the iterations for both the $\gamma$ and $\alpha$ parameters. It is evident that the algorithm converges at around 50 iterations

Before making any simulation study, the function LaplaceApproximation is employed in order to get good starting points for the independence-Metropolis algorithm. As the function LaplaceApproximation seeks point estimates, it is typically faster than any iterative simulation algorithm and provides a good independent proposal for an efficient independence sampler. For the purpose of optimization, this function offers many optimization algorithms via its argument Method. The default optimization algorithm is “SPG" which stands for Spectral Projected Gradient. It is a non-monotone algorithm that is suitable for high-dimensional models (Statisticat LLC 2015). We find that the BFGS (Broyden-Fletcher-Goldfarb-Shanno) and Nelder-Mead (NM) algorithms perform well in most of the cases. Nelder-Mead algorithm (Nelder and Mead 1965) is a derivative-free, direct search method that efficiently optimizes the low-dimensional objective functions. The advantage with the NM algorithm is that it usually converges in smaller number of iterations. It may be noted that Newton-Raphson method should be the last choice of the user as it is very sensitive to the starting values and creates problems when starting values are far from the targets. The calculation and the inversion of the Hessian matrix in this method is itself a computationally expensive task. Now, the model is fitted using Nelder-Mead as the optimization algorithm, with the following R-commands 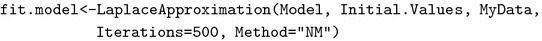 The convergence of the NM algorithm for the estimation of parameters is displayed in Fig. 3. It is seen that the algorithm converge at around 50 iterations, however, a reasonable number of iterations are needed to be specified to allow simulations.

### Summarizing output

The posterior summaries are obtained with the use of function print which prints the detailed summary. When opted for sir=TRUE, the LaplaceApproximation function returns two summaries where Summary1 summarizes the posterior modes and their corresponding posterior standard deviations of the parameters while Summary2 provides the posterior summaries based on the samples drawn with sampling importance resampling technique. Both the summaries have been tabulated in Table 2 and Table 3.

**Table 2:** Asymptotic posterior summaries along with 0.025, 0.5, 0.975 quantiles based on Laplace’s approximation

Parameter	Mode	SD	LB	UB
log.gamma	0.15	0.23	−0.31	0.60
log.alpha	3.38	0.13	3.11	3.65

**Table 3:** Posterior means and standard deviations of the parameters along with the quantiles, Monte Carlo standard errors and effective sample sizes as obtained by sampling importance resampling technique

Parameter	Mean	SD	MCSE	ESS	LB	Median	UB
log.gamma	0.07	0.23	0.01	1000.00	−0.43	0.08	0.50
log.alpha	3.40	0.14	0.00	1000.00	3.13	3.40	3.70
Deviance	115.37	1.95	0.06	1000.00	113.47	114.75	120.65

It may be noted that the results obtained are in log scale and must be exponentiated to get the values in original metric.

### Posterior analysis using simulation technique

To implement the independence-Metropolis algorithm, one needs to choose a good proposal density. The approximate posterior density which is multivariate normal returned by the function LaplaceApproximation with tabulated posterior summaries, is taken as the proposal density for the implementation of independence-Metropolis algorithm through the function LaplacesDemon. In order to have a faster convergence, firstly, the function as.initial.values is used on the object fit.model, which returns the most recent posterior samples from it. Thus, the process of updating starts from the latest values. Since, it is concerned with the simulation method involving pseudo-random number generation, it is better to set a seed so that the results can be reproduced. Finally, the model is fitted with the following set of code lines and output is summarized in Table 4
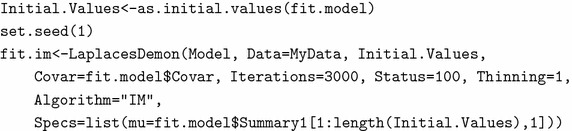
 The function LaplacesDemon returns two summary matrices of the marginal posterior distributions, one calculated over all the samples and the other calculated only on the stationary samples. However, we here report only the posterior summaries calculated on the stationary samples. The summaries include Mean which depicts posterior means of the respective parameters, SD which stands for the posterior standard deviation, MCSE (Monte Carlo standard error), ESS (effective sample size) and 2.5, 50, 97.5 % quantiles.

**Table 4:** Summarizes marginal posterior distributions of the parameters, deviance based on the MCMC samples

Parameter	Mean	SD	MCSE	ESS	LB	Median	UB
log.gamma	0.14	0.14	0.00	1149.03	−0.13	0.14	0.41
log.alpha	3.38	0.08	0.00	1243.82	3.23	3.38	3.53
Deviance	114.16	0.75	0.03	1001.04	113.44	113.94	116.17

**Fig. 4:**
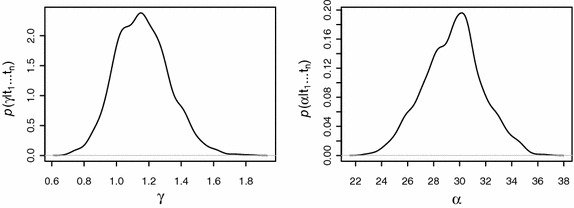
Plots of the marginal posterior densities of $\gamma$ and $\alpha$ parameters with posterior means at 1.1 and 29.6, respectively

It can be seen from Tables 2 and 4 that the posterior summaries based on simulation come out with lower standard deviation as compared to that based on Laplace approximation. This is because of two reasons. Firstly, the simulation technique summarizes posterior on the basis of samples directly drawn from it, whereas, in Laplace’s method, it is approximated asymptotically and thus, does not capture the true picture of the posterior density. Secondly, with independence-Metropolis algorithm, posterior is summarized more precisely when the proposal is a good approximation of the true posterior (Ntzoufras 2009). Having approximated the posterior with Laplace approximation and then using the approximate density as the proposal in IM algorithm, makes the posterior approximation, an excellent approximation. On the basis of MCMC samples, we plot the marginal posterior densities of the shape and scale parameters of the exponential power model as shown in Fig. 4.

**Fig. 5:**
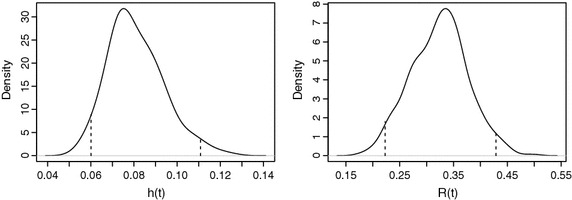
MCMC approximations to the posterior distributions of hazard rate and reliability functions at $t_{13}=23.13$. It is evident here that the posterior median of hazard rate is 0.078 with 95 % credible interval (0.06, 0.11) represented by the *dotted lines*. The reliability function has a 95 % credible region (0.22, 0.42) shown with the *dotted lines* and has median at 0.32. Moreover, the density of hazard function is right-skewed while the reliability distribution assumes a left-skewed shape

A reliability analyst is often interested in the posterior distribution of non-linear functions of the parameters, such as, reliability, failure-rate etc. Evaluation of these functions at each generated realization from the joint posterior distribution of the parameter, gives a sample from the distribution of corresponding function. Figure 5 shows the posterior distribution of reliability and failure-rate function at failure time 23.13.

## Analysis of censored data with R

A distinctive aspect of the statistical analysis of reliability data is regarding the natural occurrence of censored observations. In Bayesian set up, censoring mechanisms are easily handled as Bayesian methods take into account only the observed lifetimes and does not bother about the cause or type of censoring. Thus, for a Bayesian analyst, Type I, Type II, Type III, Type IV and random right-censoring, all correspond to the right censored data. Hence, Bayesian approach provides a common framework to analyze all censored data types.

Let $t_{\text {full}}$ denotes all the data and $t_{\text {complete},i}$ and $t_{\text {censored},j}$ represents the $i\text {th}$ complete (or uncensored) and $j\text {th}$ right-censored data respectively. Assuming that the failure times are conditionally independent given $\theta$ and that the censoring scheme is independent of the failure times, the likelihood function of all the data can be expressed as,

12 $$\begin{aligned} f(t_{\text {full}}|\,\theta )=\prod _{i=1}^{n_{\text {complete}}}f(t_{\text {complete},i}|\,\theta )\times \prod _{j=1}^{n_{\text {censored}}}[1-F(t_{\text {censored},j}|\,\theta )], \end{aligned}$$

where $\theta$ is the vector of model parameters, $n_{\text {complete}}$ and $n_{\text {censored}}$ are the number of complete and censored data respectively. Combining the above likelihood function with wisely chosen prior distributions, the analyst can define the joint posterior distribution and generate samples from it using MCMC.

In R, the analysis of any right censored data can be carried out easily by introducing a vector of binary values censor labeling uncensored observations as 1 and censored observations as 0 and listing that vector in MyData. The likelihood function will be specified according to Eq. (12). Since, we work in log-scale in LaplacesDemon, the log-likelihood LL for the right-censored data with an underlying exponential power distribution, with binary vector censor will be defined in R as following 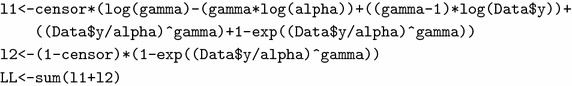


## Exponential power regression analysis

In the previous section, we considered the use of exponential power model to describe responses with no covariates (or explanatory variables). In practice, many situations involve heterogeneous populations, and to represent that heterogeneity, it is important to consider the relationship of failure time to other factors (or explanatory variables). In the present section, we focus our attention to the models containing explanatory variables namely, failure time regression models in the context of reliability.

A model with explanatory variables (or regressors) can sometimes best describe the heterogeneity in a population. It explains or predicts why some units survive a long time whereas others fail quickly. The main objective behind regression modeling is to explore the relationship between failure-time and the explanatory variables. This involves specifying a model for the distribution of $\underline{t}$, given $\underline{x}$, where $\underline{t}$ represents lifetime and $\underline{x}$ is a vector of regressor variables. It is an important class of regression models which allows one or more elements of the model parameter vector $\theta =(\theta _1, \ldots , \theta _k)$ to be a function of the regressor variables. In the present section, we develop Bayesian analysis for the non-linear regression model with random errors distributed according to the exponential power distribution. More specifically, we shall demonstrate the regression modeling of a data set in R with an underlying exponential power distribution using the LaplacesDemon package.

### Formulation of the model

Let us assume that *n* items are subject to testing and $t_1, t_2, \ldots , t_n$ be their respective observed failure times with exponential power distribution as the underlying distribution. The general idea here is to express the associated failure-time distribution as a function of a single explanatory variable $\underline{x}=(x_1, x_2, \ldots , x_n)$. Assuming the scale parameter $\alpha$ of exponential power regression model to be the function of regressor variable $\underline{x}$, the likelihood function can be expressed as

13 $$\begin{aligned} p(\underline{t}\,|\,\underline{x},\gamma ,\underline{\alpha })= \bigg (\frac{\gamma }{(\alpha (x_i))^\gamma }\bigg )^n\prod _{i=1}^n\big (t_i\big )^{\gamma -1}\text {exp} \bigg [\sum _{i=1}^n\bigg ({\frac{t_i}{\alpha (x_i)}}\bigg )^\gamma \bigg ]\text {exp}\bigg (n-{\sum _{i=1}^n}e^{(t_i/\alpha (x_i))^\gamma }\bigg ) \end{aligned}$$

A variety of functional forms, technically known as *link function* for $\alpha (x)$, are often employed but the most useful form is perhaps the log-linear one for which

$$\begin{aligned} \log (\alpha (x_i))&= \beta _0+\beta _1x_i \end{aligned}$$

Working in the log scale, we have log-likelihood function as

14 $$\begin{aligned}&\log {p(\underline{t}\,|\,\underline{x}, \beta _0, \beta _1, \gamma)}=n\log {\gamma }-\gamma  \sum\limits_{{i = 1}}^{n} {(\beta _{0}  + \beta _{1} x_{i} )}  +(\gamma -1) \sum\limits_{{i = 1}}^{n} {\log (t_{i} )}  \nonumber \\&+\sum_{i=1}^n\bigg ({\frac{t_i}{\exp (\beta _0+\beta _{1}x_i)}}\bigg )^\gamma +n-{\sum_{i=1}^n}e^{(t_i/{\exp (\beta _0+\beta _{1}x_i)})^\gamma } \end{aligned}$$

The unknown regression coefficients $\beta _0$ and $\beta _1$ replace the scale parameter $\underline{\alpha }$ in the model. We now proceed to specify priors on $\beta$’s and shape parameter $\gamma$. Again opting for unconstrained optimization for the positive parameter discussed in Subsection “Model specification”, we put a half-Cauchy prior distribution on $\gamma$. Each of the $\beta$ parameters are assigned weakly-informative normal prior distributions with large standard deviations, indicating a lot of uncertainty about them. Thus, we have,

15 $$\begin{aligned} \begin{aligned} \gamma \sim \text {half-Cauchy}(25)\\ \beta _j \sim \text {N}(0,1000)\\ \end{aligned} \end{aligned}$$

The complex log-likelihood function itself suggests a non-standard joint posterior distribution thereby making the analytic solution, a difficult task. However, the same posterior analysis can be carried out easily in R using the functions of LaplacesDemon package. Progressing the same way, we first enter the data set in the listed format in R followed by the model specification and choosing guess values for the model parameters. To display the efficiency of the written R-codes for the regression analysis, we choose a data set with known parameter values obtained through simulation technique. Using a random-seed 1, we simulate a single regressor variable $\underline{x}$ of length 15 from Beta(1, 1) distribution. Fixing $\gamma =1, \beta _0=1$ and $\beta _1=2$, we finally obtained a data set of length 15 from the exponential power regression model. The fitting of the model is done with the following set of code lines: 
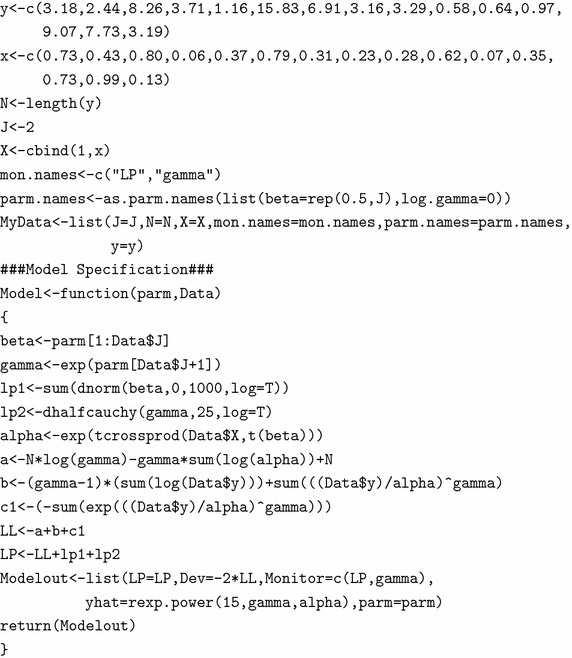


### Initial values

With no prior knowledge, it is a good idea to either set all of the initial values to zero or to randomize each initial value using GIV function 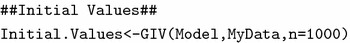


### Posterior analysis by Laplace’s method

The Laplace’s method is implemented via the function LaplaceApproximation. The optimization algorithm selected in this case is NM. However, the user may go for BFGS algorithm also 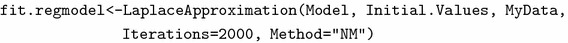


### Summarizing output

The relevant posterior summaries are obtained with the use of function print and is tabulated in Table 5 and Table 6. When opted for sir=TRUE, the function LaplaceApproximation returns the posterior modes and standard deviations of the parameters in Summary1 whereas Summary2 provides the posterior summaries based on samples drawn with sampling importance resampling technique.

**Table 5:** Posterior summaries using the function LaplaceApproximation

Parameter	Mode	SD	LB	UB
beta[1]	1.13	0.25	0.63	1.62
beta[2]	1.69	0.45	0.79	2.59
log.gamma	0.17	0.23	−0.28	0.62

**Table 6:** Posterior means and standard deviations of the exponential power parameters based on the samples drawn by sampling importance resampling algorithm

Parameter	Mean	SD	MCSE	ESS	LB	Median	UB
beta[1]	1.20	0.27	0.01	1000.00	0.68	1.18	1.78
beta[2]	1.57	0.51	0.02	1000.00	0.62	1.58	2.55
log.gamma	0.03	0.24	0.01	1000.00	−0.46	0.05	0.48
Deviance	71.93	3.36	0.11	1000.00	68.75	71.04	83.87

It follows from Table 5 that the posterior modes of the regression parameters $\beta _0$ and $\beta _1$ for the concerned model is $1.13\pm 0.25$ and $1.69\pm 0.45$, respectively. For more accurate summary, we resort to simulation technique.

### Simulation-based study

The function LaplacesDemon is used to simulate from the posterior density. The fitting of the model is done and output is summarized in Table 7: 
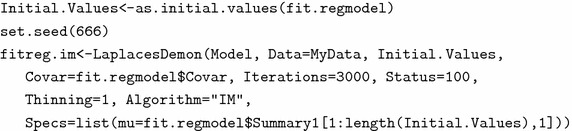

**Table 7:** Marginal posterior summaries based on the MCMC samples using independence-Metropolis algorithm

Parameter	Mean	SD	MCSE	ESS	LB	Median	UB
beta[1]	1.13	0.15	0.01	890.36	0.85	1.13	1.43
beta[2]	1.68	0.26	0.01	779.53	1.18	1.68	2.21
log.gamma	0.15	0.13	0.01	815.56	−0.10	0.15	0.41
Deviance	69.52	0.86	0.05	574.48	68.58	69.30	71.68

**Fig. 6:**
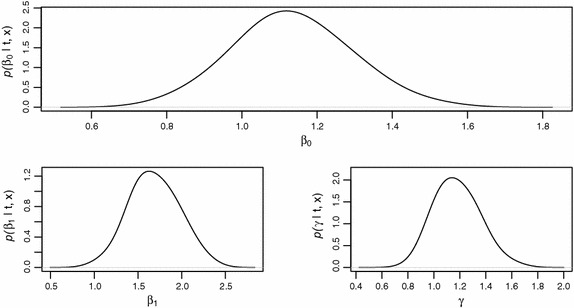
Marginal posterior densities of regression coefficients $\beta _0$ and $\beta _1$ and shape parameter $\gamma$ with their posterior mean values as 1.1, 1.7 and 1.1, respectively

The posterior means for the regression parameters on the basis of MCMC samples are $1.13\pm 0.15$ and $1.68\pm 0.26$ respectively. The reduced posterior standard deviations for the parameters based on MCMC (IM) posterior samples as reported in Table 7, depict a precise posterior approximation. The marginal posterior densities of the three parameters of the exponential power regression model are displayed in Fig. 6.

## Determination of burn-in and replacement time

A bathtub curve are useful in reliability related decision making. Reducing the burn-in time of a new product with too high initial failure rate results in improved reliability of the product. Similarly, during the wear-out phase of the product, the failure increases rapidly and replacement is needed to reduce the risk of immediate failure. The problem of determining burn-in time and replacement time can easily be tackled by the failure-rate criteria (Xie and Lai 1996).

Suppose that the product can only be released after time when the failure rate is lower than $r_b$ to meet customer’s requirement, then the optimum burn-in time can be determined by the solving the following equation numerically using any standard algorithm:

$$\begin{aligned} (\gamma /(\alpha )^\gamma )\,t^{\gamma -1}\,\text {exp}[(t/{\alpha })]^\gamma =r_b \end{aligned}$$

As the shape of the failure-rate curve suggests, there will be two solutions to the above equation and the optimum burn-in time will be the smallest *t* for which the equality holds.

Similarly, suppose that the criterion for the replacement of the product is that the failure rate must not be higher than the acceptable level $r_c$. Then, the replacement time can be obtained by solving the following equation:

$$\begin{aligned} (\gamma /(\alpha )^\gamma )\,t^{\gamma -1}\,\text {exp}[(t/{\alpha })]^\gamma =r_c \end{aligned}$$

where, *t* is the time by which the product should be replaced. The largest of the two solutions will be the optimum replacement time for the product as the the failure rate is increasing and higher than the acceptable level after this time.

## Real data modeling

The R codes are used to model two real data sets and the most relevant results are reported.

### Electronic device failure time data

Wu et al. (2005) presented a dataset consisting of 18 lifetime observations of an electronic device: 5, 11, 21, 31, 46, 75, 98, 122, 145, 165, 195, 224, 245, 293, 321, 330, 350, and 420. The same dataset has been considered by several authors to fit different bathtub distributions. We apply the R codes developed in Section “Bayesian analysis of exponential power model” to model this data set with an underlying exponential power distribution. On the basis of 2000 MCMC simulations, posterior means of shape and scale parameters are found to be $\gamma =0.911$ and $\alpha =273.52$ with posterior standard deviations as 1.132 and 1.09, respectively. The respective 95 % credible intervals for $\gamma$ and $\alpha$ are,

$$\begin{aligned} \gamma \in (0.721, 1.17)\, \, \text {and} \,\, \alpha \in (230.14, 326.033) \end{aligned}$$

**Fig. 7:**
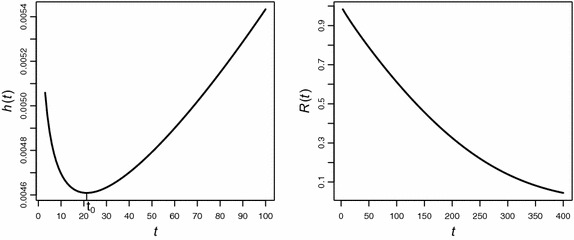
The *left panel* of the graph displays the hazard curve with a changing time point at $t_0=21.29$ while the *right panel* shows decreasing reliability of the device with the passage of time

The deviance for the model has been found to be equal to 219.42. Figure 7 gives the graphical representation of the reliability and hazard curve for this data set.

### Transistor data

**Fig. 8:**
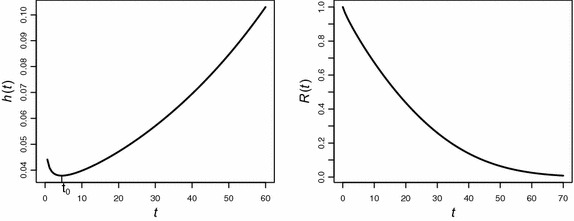
The *left panel* of the graph displays the hazard curve assuming a bathtub-shape attaining minimum value at $t_0=4.52$ while the *right panel* depicts decreasing reliability of the transistors with changing time

Wilk et al. (1962) give data on the lifetimes (in weeks) of 34 transistors in an accelerated life test. Three of the times are censoring times and are denoted by asterisks: $3, 4, 5, 6, 6, 7, 8, 8, 9, 9, 9, 10, 10, 11, 11, 11, 13, 13, 13, 13, 13, 17, 17, 19, 19, 25, 29, 33, 42, 42, 52, 52^{*}, 52^{*}, 52^{*}$. This data set has also been considered by Lawless (1982) under the assumption of gamma model. The R codes suggested in Section “Analysis of censored data with R” are used to fit this data set to exponential power model. Based on 3000 MCMC simulations, the posterior mean value of the shape parameter $\gamma$ is found to be equal to 0.85 with posterior standard deviation as 1.09 whereas, the posterior mean of scale parameter $\alpha$ is 36.12 with standard deviation as 1.08. The 95 % credible intervals for the respective parameters are (0.71, 1.01) and (30.84,41.86) respectively. The deviance for the exponential power model is 254.39. The graphical display of hazard and reliability curves are provided in Fig. 8.

## Discussion and conclusion

This paper develops the Bayesian inference procedures for the exponential power model assuming weakly-informative priors for the model parameters. This parsimonious model with just two-parameters is fairly applicable to various real-life failure-time data capable of producing increasing as well as bathtub-shaped failure rate. These two properties along with the availability of invertible cumulative distribution function makes the exponential power model, a useful alternative to the conventional Weibull distribution. A distinguishing feature of this paper is that both the analytic and simulation-based Bayesian studies are conducted in R language using the package LaplacesDemon. The main body of the manuscript contains the complete description of R codes both for the null and regression models with random errors distributed according to the exponential power distribution. Illustrations have been made using simulated data sets which is finally concluded on real-world reliability problems. The posterior means, modes and 95 % credible intervals for the parameters are obtained. The exact posterior densities of the parameters together with that of hazard and reliability functions are plotted. It is seen that, the two functions LaplaceApproximation and LaplacesDemon exploited throughout the paper, allow fast and precise posterior analysis. However, since, LaplaceApproximation is asymptotic in nature, it should be noted that the sample size is at least 5 times the number of parameters, in order to observe its good performance. Simulation tools are free from such restrictions. Furthermore, it has been observed throughout that the simulation technique, particularly, independence-Metropolis algorithm summarizes the posterior more pecisely, in terms of the lower standard deviations of the parameters. However, it is to be noted that, IM algorithm performs well if the proposal is a good approximation of the posterior. Therefore, the posterior approximation using Laplace approximation can always be improved with independence-Metropolis algorithm.

## Limitations of the study

The Bayesian study has been carried out only for complete and right censored data. The case of left-censored and interval-censored data are yet to be considered.
